# Improving T3 Medication Authorisation Compliance on an Old Age Psychiatry Ward: A Quality Improvement Project

**DOI:** 10.1192/bjo.2026.11399

**Published:** 2026-06-30

**Authors:** Ishan Jain, Cara Webb

**Affiliations:** Hollins Park Hospital, Warrington, United Kingdom

## Abstract

**Aims:**

Unlawful medication administration under the Mental Health Act (MHA) poses significant patient safety and legal risks in Old Age Psychiatry. Cognitive impairment, fluctuating capacity and behaviour disturbances increase the likelihood of patients requiring rapid tranquillisation. In August 2025, four incidents occurred, where either the route or frequency were administered outside the parameters of medication authorisation forms (T3 particularly).

This Quality Improvement Project aimed to eliminate T3 medication errors by improving Registered Nurses' (RN) knowledge and confidence in compliance with MHA medication authorisation forms. We hypothesised that a combined intervention of a visual prompt and targeted teaching would improve compliance and reduce errors to zero.

**Methods:**

We included all RNs on a 16-bed Old Age Psychiatry ward (n=10). Baseline data included incident reports and questionnaires identifying inconsistent knowledge and varied confidence of medication form processes among RNs.

Two interventions were implemented: (1) a visual whiteboard placed above the drug trolley identifying patients with active T3 medication forms to prompt checks at administration times; (2) a targeted teaching session using real ward-based incidents to reinforce legalrequirements for T3 authorisation. Monthly audits monitored medication authorisation errors, and questionnaires were repeated following the teaching session.

**Results:**

Following implementation, off-T3 medication incidents reduced from four in August 2025 to zero in September 2025, and remained at zero for four consecutive months. Staff reported that the visual prompt aided adherence to MHA T3 medication forms. Mean knowledge questionnaire scores increased from 58% at baseline to 71% after the teaching session. Mean self-reported confidence in interpreting T3 forms increased from 3.7 to 4.3 on a 5-point Likert scale.

**Conclusion:**

Low-cost, scalable interventions integrating visual aids and focussed education eliminated unlawful T3 medication errors to zero and significantly improved staff knowledge and confidence, ensuring sustainable T3 compliance. This Quality Improvement approach supports safer practice and is transferrable to other inpatient psychiatric services.

